# Exogenous Brassinolide Alleviates Salt Stress in *Malus hupehensis* Rehd. by Regulating the Transcription of NHX-Type Na^+^(K^+^)/H^+^ Antiporters

**DOI:** 10.3389/fpls.2020.00038

**Published:** 2020-02-06

**Authors:** Qiufang Su, Xiaodong Zheng, Yike Tian, Caihong Wang

**Affiliations:** ^1^College of Horticulture, Qingdao Agricultural University, Qingdao, China; ^2^Qingdao Key Laboratory of Genetic Improvement and Breeding in Horticulture Plants, Qingdao Agricultural University, Qingdao, China

**Keywords:** brassinolide, oxidative stress, plant hormone, ion homeostasis, *MhNHXs*

## Abstract

Brassinolide (BL) mediates various physiological processes and improves plant tolerance to abiotic stresses. However, the effects and mechanism of exogenous BL on the salt tolerance of apple seedlings remain unclear. Herein, we investigated the role of BL in the salt stress response of *Malus hupehensis* Rehd., a widely grown apple rootstock. Salt-stressed apple seedlings showed significant decline in chlorophyll content and photosynthetic rate, and the application of 0.2 mg/L exogenous BL alleviated salt stress and maintained photosynthetic capacity. Exogenous BL application can strengthen the activities of superoxide dismutase and catalase and thereby eliminates reactive oxygen species (ROS) production induced by salt stress and promote the accumulation of proline and soluble sugar, thus maintaining osmotic balance. Furthermore, exogenous BL application decreased Na^+^ accumulation and increased K^+^ content in shoots and roots under salt stress by regulating the expression levels of Na^+^(K^+^)/H*^+^* antiporter genes (*MhNHXs*). MhBZR1 and MhBZR2, which are the key transcription factors in the BR signal transduction pathway, can directly bind to the promoter of *MhSOS1* and *MhNHX4-1*, respectively, and inhibit their expression. Our findings would provide a theoretical basis for analyzing the mechanism of exogenous BL application on the salt tolerance of apples.

## Introduction

Soil salinity is a major type of abiotic stress that limits crop productivity and affects plant growth and development. More than 800 million hectares of land is affected by salinity, and this problem continues to worsen (Liang et al., 2018; Yang and Guo, 2018; Feng et al., 2019). Apples (*Malus domestica* Borkh.) are one of the most valuable horticultural fruit crops cultivated worldwide. The growth and productivity of apple trees is severely limited by salt stress in China, especially in the Yellow River Delta. The negative effects of salt stress on apple trees, as perennial crops, not only are limited to the current year but also extends to subsequent years (Navarro et al., 2003; An et al., 2018). Therefore, strategies for improving the salt tolerance of apple trees should be explored.

Under salt stress conditions, plants are typically stressed in three ways: osmotic stress, ionic stress, and oxidative damage (Yang and Guo, 2018). A low water potential causes water deficits in roots (Hasegawa et al., 2000). Excessive external and internal Na^+^ accumulation in the cytoplasm disrupts the homeostasis of Na^+^:K^+^ and evokes toxic ion effects. Oxidative damage is usually triggered by osmotic and ionic stress. Given that plants are sessile, they are inevitably affected by salt stress. After prolonged adaptation, plants gradually evolve a precise mechanism as a response to salt stress (Sun et al., 2015a; Mao et al., 2017). For example, a series of salt-responsive genes involved in salt stress signal transduction are activated in response to salt stress (Lopez-Gomez et al., 2016). Reactive oxygen species (ROS) induced by salt stress is alleviated by antioxidant systems, including antioxidants and antioxidant enzymes (Tofighi et al., 2017; Wu et al., 2017). The accumulation of osmolytes, such as sorbitol, proline, and lycine, is critical to osmotic balance (Shahid et al., 2014; Yusuf et al., 2017). Maintaining homeostasis of Na^+^ and K^+^ in cells by activating Na^+^ and K^+^ transporters is vital for plant salt stress tolerance (Azhar et al., 2017).

Cytosolic Na^+^:K^+^ ratio is one of the key features conferring salinity stress tolerance in plants, and this trait is often considered a potential screening tool for plant breeders (Poustini and Siosemardeh, 2004). The salt overly sensitive (SOS) pathway, which is essential for maintaining Na^+^ and K^+^ homeostasis in the cytoplasm and defense against salt stress, has been well identified and characterized (Zhu, 2002; Zhu, 2016). The SOS pathway is relatively specific for ionic transportation, in which high Na^+^ stress initiates a calcium signal that stimulates the SOS3–SOS2 protein kinase complex, which then activates the Na^+^/H^+^ exchange transporter SOS1, resulting in the exclusion of excess Na^+^ from plant cells, thereby enhancing salt detoxification (Qiu et al., 2004; Hu et al., 2016a). *SOS1* belongs to the NHX-type Na^+^(K^+^)/H^+^ exchange family genes (*NHXs*), and the NHXs family is essential for Na^+^ and K^+^ balance in plants.

NHX proteins serve a primary role in ion homeostasis, which is an essential cellular process for salt stress response. The *Arabidopsis* genome encodes eight NHX homologs that have been grouped based on sequence similarities and functions into three distinct classes, namely, plasma membrane (NHX7 and NHX8), endosomal, or vesicular (NHX5 and NHX6; Bassil et al., 2011a; Bassil et al., 2011b), and into four vacuolar homologs, namely, NHX1, NHX2, NHX3, and NHX4 (Leidi et al., 2010). In apple trees, the overexpression of *MdSOS1* can enhance salt tolerance and reduce Na^+^ content (Liu et al., 2012). Besides transport of Na^+^, AtNHX1 and AtNHX2 are reported to control vacuolar K^+^ homeostasis to regulate growth, flower development, and reproduction (Bassil et al., 2011a), and they are essential for active K^+^ uptake in the tonoplast for stomatal function (Barragan et al., 2012).

Plant hormones are essential endogenous molecules and signals involved in regulating plant development and tolerance to diverse stresses, including salinity stress (Ryu and Cho, 2015). The application of plant growth regulators, such as gibberellin 3 (GA3), salicylic acid (SA), and jasmonic acid (JA), is an effective approach for improving the salt tolerance of crops (Shahzad et al., 2018). Brassinolide (BL), as a highly active synthetic analog of brassinosteroids (BRs), play numerous important roles in plant growth and development (Clouse, 1996; Kim et al., 2009). The exogenous application of BL also has major effects on plants and mitigates the adverse effects of salt stress (Yusuf et al., 2012). Exogenous BL application can improve photosynthetic efficiency in different plant species under salt stress by increasing the levels of hormones, especially ZR, iPA, IAA, and SA, increasing gas exchange, enhancing antioxidant enzyme activities, and delaying plant senescence (Yusuf et al., 2012; Wu et al., 2017). In addition, exogenous BL can increase the protein levels and the expression of various salt-responsive genes to different levels (Sharma et al., 2013), but the mechanisms in which exogenous BL application induces the expression of salt-responsive genes remain largely unexplored.

Over the past decade, the BR signaling pathway has been explored in plants (Wang et al., 2012; Ye et al., 2012). In the presence of BRs, brassinosteroid-insensitive 2 (BIN2) is dephosphorylated by bri1-suppressor 1 (BSU1) and degraded by the 26S proteasome, which subsequently suppresses brassinazole resistant1 (BZR1) and bri1-ems-suppressor1 (BES1)/BZR2 by BIN2 (Yin et al., 2002). BZR1 and BES1 are the key transcription factors in the BR signaling pathway, and dephosphorylated BZR1 and BES1 can directly bind the E-BOX (CANNTG) and BRRE elements (CGTGT/CG) of the BR-related gene promoters to regulate the expression of these genes (Shahnejat-Bushehri et al., 2016). However, the molecular mechanism in which BZR1 and BES1 responds to salt stress remain unknown.

Apple trees are often subject to severe salt stress, resulting in a remarkable loss of apple production and quality. Several agronomic practices are employed to mitigate the adverse effects of salt stress. The exogenous application of BL can improve plant tolerance to salt stress in rice and tomato. However, the effects and mechanism by which BL affects the tolerance of woody plants to salt stress are not well documented. In the present study, we investigated the effects of different concentrations of exogenous BL on apple seedlings under salt stress. Then, we explored the potential physiological and molecular mechanisms by which BL influences antioxidative activity, osmotic balance, and ion homeostasis. This finding will enhance the applications and examination of the physiological role of BL under salt stress.

## Materials and Methods

### Plant Materials and Growth Conditions

Apple seeds (*Malus hupehensis*) were sown in wet vermiculite after cold stratification. Apple seedlings were grown at 23 ± 2°C in a 16/8 h light/dark cycle. Light intensity was approximately 100 µmol·m^−2^·s^−1^. Apple calli induced from “Orin” embryos were grown on MS medium containing 1.5 mg·L^–1^ 2,4-dichlorophenoxyacetic acid and 0.4 mg·L^–1^ 6-BA at 23 ± 2°C in darkness. The calli were subcultured every 2 weeks.

### Salt Stress and Exogenous BL Treatment

One hundred eighty 1-month-old apple seedlings were randomly divided into five groups. The apple seedlings in group I were watered with a complete nutrient solution as the control, and group II was treated with 200 mM NaCl. Except for the same salt stress with group II, the seedlings in groups III, IV, and V were sprayed with 0.05, 0.2, and 1.0 mg/L of BL, respectively. BL (Solarbio, Beijing, China) was sprayed every 2 days. Each experiment was independently repeated three times.

### Measurements of Chlorophyll Content and Photosynthetic Rate

For determination of chlorophyll content, a SPAD-502 chlorophyll meter (Konica Minolta, Tokyo, Japan) was used. The photosynthetic rate was measured using a LI-6400XT meter (LI-COR, Lincoln, USA). The light intensity was set at 500 µmol·m^−2^·s^−1^ at an approximately 50% humidity. The temperature was set at 22°C.

### Measurements of ROS Levels and Malondialdehyde Content

H_2_O_2_ and O_2_·^−^ staining was conducted as described by Zheng et al. (2017). The malondialdehyde (MDA) content of the leaves was detected using a plant MDA extraction kit (Nanjing Jiancheng Bioengineering Institute, Nanjing, China).

### Detection of Antioxidant Enzyme Activities

A total of 0.1 g of leaves from apple seedlings were used for the detection of antioxidant enzyme activities. Superoxide dismutase (SOD), peroxidase (POD), and catalase (CAT) activities were detected according to the plant SOD, POD, and CAT extraction kits (Nanjing Jiancheng Bioengineering Institute, Nanjing, China) following the manufacturer’s instructions.

### Determination of Electrolyte Leakage and Osmolytes

A total of 0.5 g of leaves from apple seedlings were used for the detection of electrolyte leakage and osmolytes. Electrolyte leakage was determined as described previously (Ahmad et al., 2016). The proline content was determined as described by Wani et al. (2017). The absorbance was obtained at 520 nm by using a spectrophotometer (Yoke, Shanghai, China). Soluble protein and soluble sugar contents were determined as described by Qiu et al. (2019) and Sharma et al., 2019, respectively.

### Determinations of Na^+^ and K^+^

A total of 0.5 g (dry weight) of shoots and roots from apple seedlings were used for the detection of Na^+^ and K^+^ contents. The concentrations of Na^+^ and K^+^ were measured by inductively coupled plasma-optical emission spectrometry (PerkinElmer, Waltham Massachusetts, USA) as described by Liang et al. (2017).

### Quantitative Reverse Transcription Polymerase Chain Reaction Assay

Total RNA extraction and quantitative polymerase chain reaction (qPCR) assay were conducted as described by Zheng et al. (2017). *MhActin* (accession number: MDP0000774288) was used as an internal control. Primer sequences for qPCR were designed according to the coding sequence of *MhNHXs* and *MhBZRs* by using the Primer 5 software and checked using BLAST search in the apple genomic database. The primer sequences are shown in **Table S1**.

### Cloning of *MhBZRs* Genes and the Prokaryotic Expression of Their Proteins in *Escherichia coli*

The MhBZR1 (MDP0000157809), MhBZR2 (MDP0000306427), MhBZR3 (MDP0000203462), MhBZR4 (MDP0000218271), MhBZR5 (MDP0000344348), and MhBZR6 (MDP0000130173) coding regions were cloned from *M. hupehensis*. The coding sequences of *MhBZRs* were amplified and ligated into the pGEX-6P-1 vector containing a GST tag. The primers are shown in **Table S1**. The GST-MhBZRs fusion proteins were expressed in *E. coli* (BL21) and purified using glutathione-sepharose 4B (GE Healthcare, Little Chalfont, UK).

### Electrophoretic Mobility Shift Assay

Electrophoretic mobility shift assays (EMSA) were employed to detect a combination of the GST-MhBZRs proteins with the promoters of *MhNHXs* in which the *MhNHXs* promoter fragments containing the E-box (CANNTG) were produced using biotin-labeled (Invitrogen, Shanghai, China) or unlabeled oligonucleotide (to be used as a competitor). The primers are shown in **Table S1**. The EMSA reaction was performed as described by Zheng et al. (2018).

### Detection of GUS and LUC Expression Assays in the Transiently Transformed Apple Calli

The *MhSOS1* and *MhNHX4-1* promoters were cloned from *M. hupehensis* and ligated into the pCAMBIA1301 vector, and the coding sequences of MhBZR1/MhBZR2 were amplified and ligated into the pMDC83 vector. The primers are shown in **Table S1**. *35S::MhBZR1*/*MhBZR2-GFP*, *proMhSOS1*/*MhNHX4-1::GUS* (β-glucuronidase), and *35S::LUC* were transiently transformed together into the *Agrobacterium* strain EHA105 for the transformation of apple calli. EHA105 carrying *35S::GFP*, *proMhSOS1*/*MhNHX4-1::GUS*, and *35S::LUC* was used as the control. The transient transformation of apple calli and the GUS and LUC expression assays were performed as described previously (Zheng et al., 2018).

### Experimental Design and Statistical Analysis

All experiments were repeated three times according to a completely randomized design. The data were analyzed by ANOVA followed by Fisher’s LSD or Student’s *t-*test analysis. Statistically significant differences were indicated by *P* < 0.05. Statistical computations were conducted using SPSS (IBM, Armonk, NY, USA).

## Results

### Exogenous BL Treatment Improved the Salt Tolerance of Apple Seedlings

**Figure 1:**
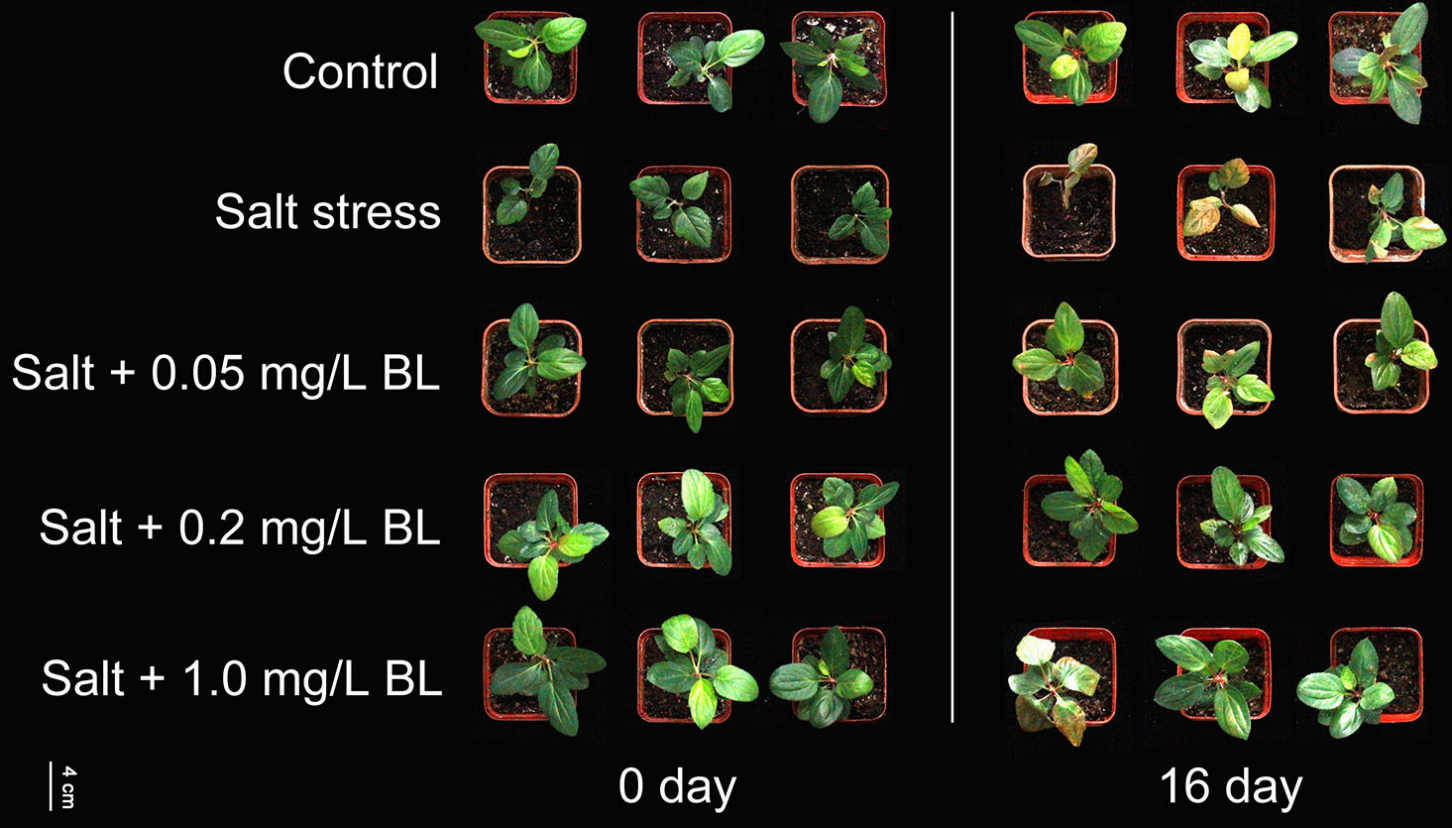
The phenotype resulting from the application of different concentrations of exogenous brassinolide (BL) (0.05, 0.2, and 1.0 mg/L) to *Malus hupehensis* under 200 mM NaCl stress at day 0 and day 16. Exogenous BL was sprayed on the leaves of apple seedlings every 2 days. Each experiment was independently repeated three times.

BL at different concentrations was sprayed on the leaves of *M. hupehensis* seedlings under 200 mM NaCl stress. As shown in **Figure 1**, the apple seedlings were seriously damaged, showing withered and chlorotic leaves under salt stress. At low (0.05 mg/L) and high BL concentrations (1.0 mg/L), the growth vigor of the seedlings was much better than those without BL treatment, but the leaves still withered noticeably. However, when 0.2 mg/L BL was applied to the apple seedlings, the growth of the seedlings under salt stress was similar to that of the control seedlings under normal conditions. Therefore, 0.2 mg/L BL was selected for further experiments.

### Effects of Exogenous BL Application on Chlorophyll Content and Photosynthetic Rate Under Salt Stress

**Figure 2:**
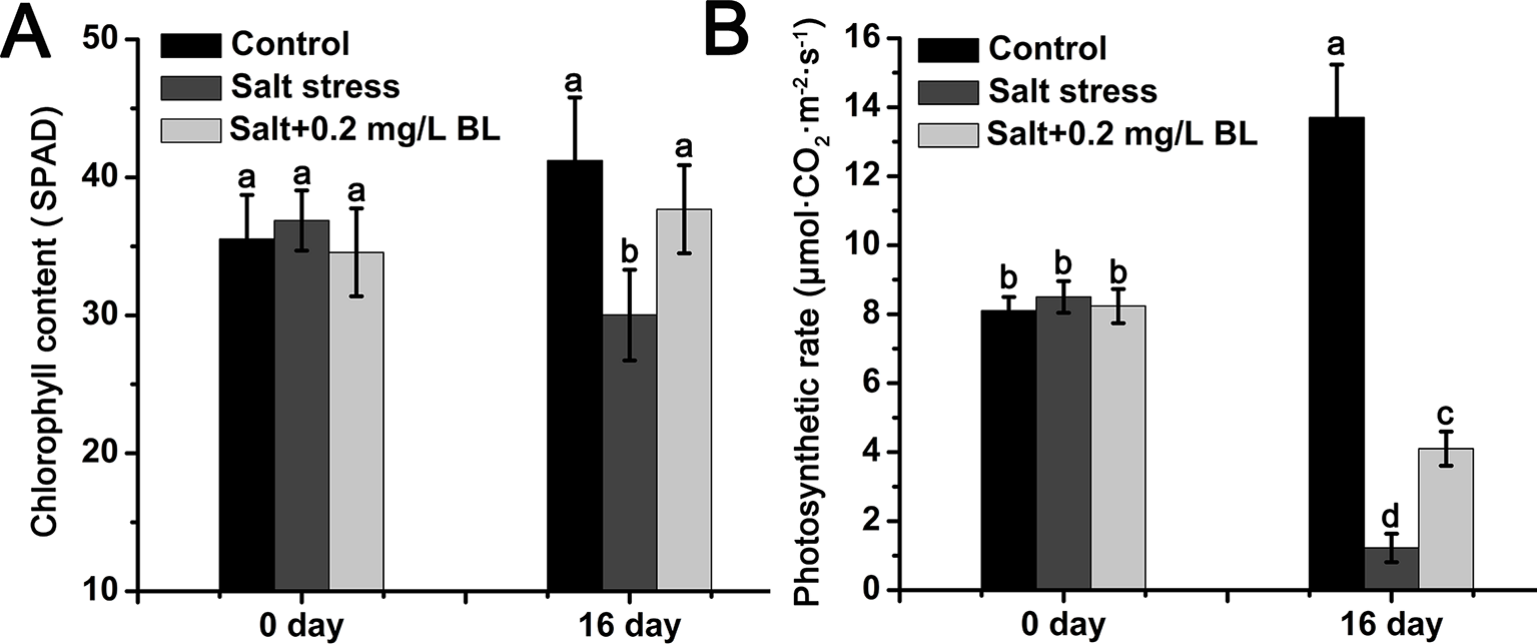
Effects of exogenous BL application on the chlorophyll content **(A)** and photosynthetic rate **(B)** of apple seedlings before and after salt stress. Each experiment was independently repeated three times. Data represent the means ± SD of three biological replicates. Different lowercase letters indicate significant differences, according to Fisher’s LSD (*P* < 0.05).

As shown in **Figure 2A**, salt stress dramatically reduced the chlorophyll content from 41.2 SPAD to 30.0 SPAD but increased chlorophyll content to 37.6 SPAD upon exogenous BL application. The photosynthetic rate of apple seedlings sharply decreased from 8.0 to 1.2 µmol·m^−2^·s^−1^ under salt stress. When exogenous BL was applied, the photosynthetic rate of the apple seedlings reached 4.1 µmol·m^−2^·s^−1^ (**Figure 2B**).

### Effects of Exogenous BL Application on Oxidative Damage and Antioxidant Enzyme Activities Under Salt Stress

**Figure 3:**
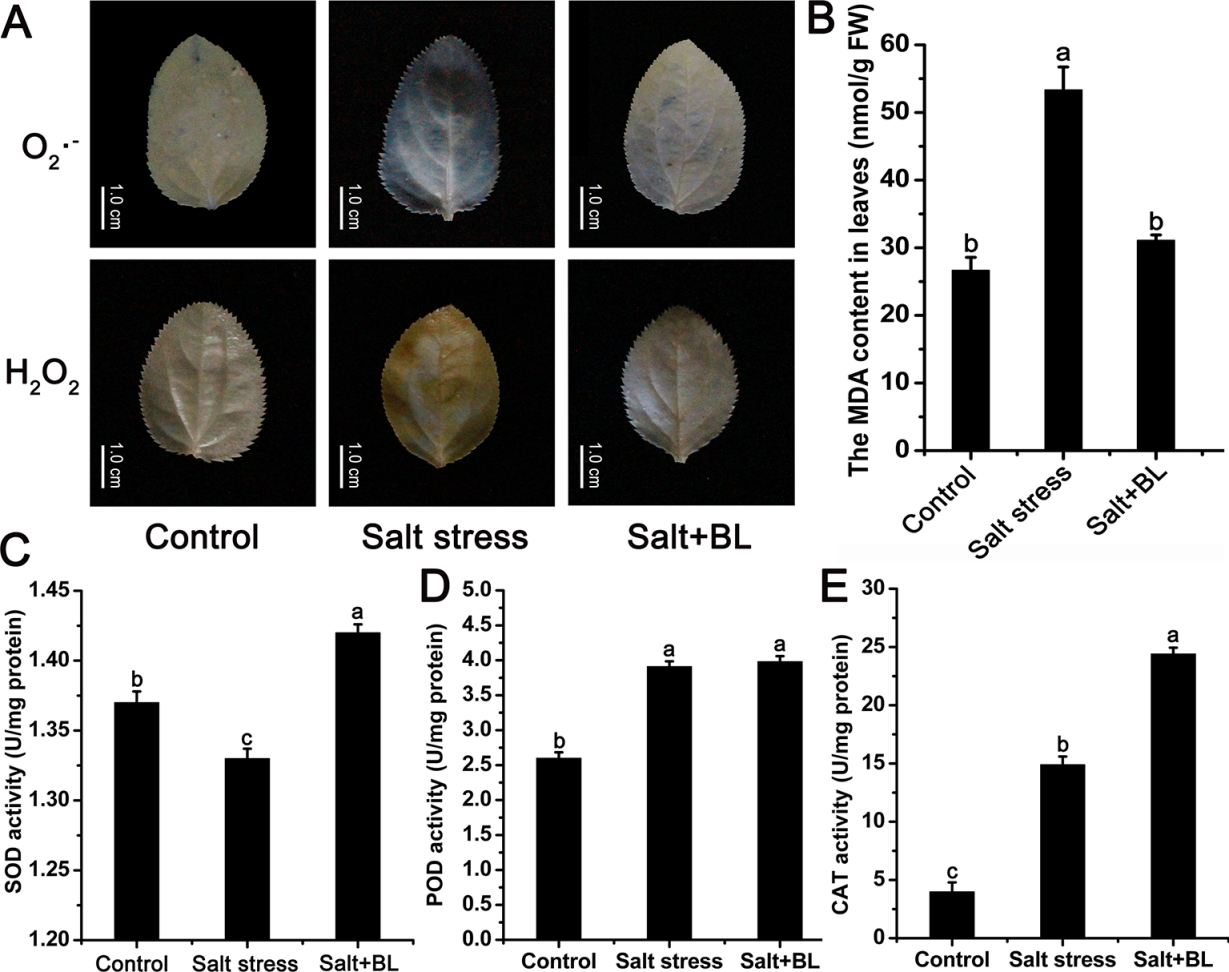
Effects of exogenous BL application on the levels of O_2_·^−^ and H_2_O_2_ **(A)** and malondialdehyde (MDA) contents **(B)** under salt stress. Effects of exogenous BL application on the activities of superoxide dismutase (SOD) **(C)**, peroxidase (POD) **(D)**, and catalase (CAT) **(E)** under salt stress. The scale bars in **(A)** represent 1.0 cm. Each experiment was independently repeated three times. Data represent the means ± SD of three biological replicates. Different lowercase letters indicate significant differences, according to Fisher’s LSD (*P* < 0.05).

Salt stress significantly elevated the O_2_·^−^ and H_2_O_2_ levels in the leaves of apple seedlings, whereas exogenous BL significantly reduced these levels (**Figure 3A**). Consistently, the MDA content under salt stress was twice that of the control group. When exogenous BL was applied, the MDA content recovered to the control level (**Figure 3B**).

The activities of antioxidant enzymes SOD, POD, and CAT were measured. SOD activity was 1.33 U/mg protein under salt stress. When exogenous BL was applied, SOD activity increased to 1.42 U/mg protein (**Figure 3C**). CAT activity was only 14.9 U/mg protein after salt stress, and exogenous BL treatment increased CAT activity to 24.4 U/mg protein (**Figure 3E**). During POD activity, no difference was observed in the seedlings treated with or without exogenous BL (**Figure 3D**).

### Effects of Exogenous BL Application on the Electrolyte Leakage and Osmolytes Under Salt Stress

**Figure 4:**
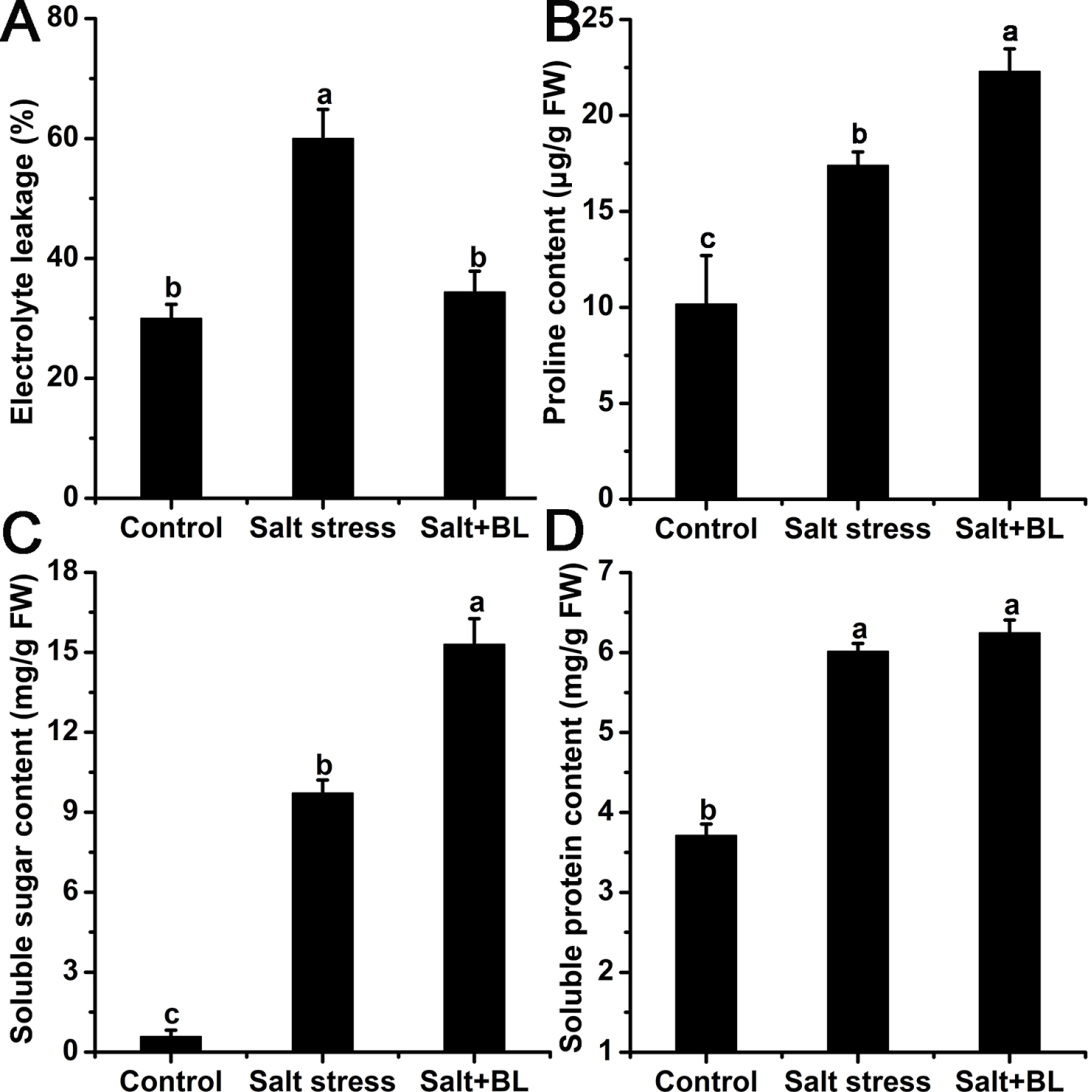
Effects of exogenous BL application on electrolyte leakage **(A)**, proline content **(B)**, soluble sugar content **(C)**, and soluble protein content **(D)** under salt stress. Each experiment was independently repeated three times. Data represent the means ± SD of triplicate experiments. Different lowercase letters indicate significant differences, according to Fisher’s LSD (*P* < 0.05).

As shown in **Figure 4A**, electrolyte leakage was sharply induced by salt stress but was reduced by 43.18% compared with that in the salt-treated plants alone when exogenous BL was applied.

We measured the contents of osmolytes, including proline, soluble sugar, and soluble protein. The contents of proline, soluble sugar, and soluble protein were all significantly induced by salt stress (**Figure 4**). When exogenous BL was applied, the contents of proline and soluble sugar were increased compared with the apple seedlings under salt stress, and the content of soluble protein had no change.

### Effects of Exogenous BL Application on the Na^+^ and K^+^ Contents Under Salt Stress

**Figure 5:**
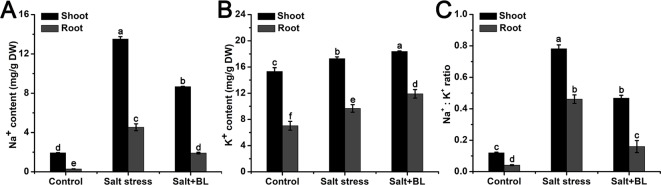
Effects of exogenous BL application on Na^+^ content **(A)**, K^+^ content **(B)**, and the Na^+^:K^+^ ratio **(C)** in shoots and roots under salt stress. Each experiment was independently repeated three times. Data represent the means ± SD of three biological replicates. Different lowercase letters indicate significant differences, according to Fisher’s LSD (*P* < 0.05).

The Na^+^ and K^+^ contents in the shoots and roots were measured. As shown in **Figure 5A**, the content of Na^+^ was sharply induced by salt stress both in the shoots and roots. When exogenous BL was applied, the Na^+^ content decreased significantly. The K^+^ content increased in the shoots and roots due to salt stress and exogenous BL (**Figure 5B**). The Na^+^:K^+^ ratio was significantly increased under salt stress, and exogenous BL reduced sodium concentrations, increased potassium concentrations, and reduced the Na^+^:K^+^ ratio under salt stress in the shoots and roots (**Figure 5C**).

### Effects of Exogenous BL Application on the Expression of the *MhNHXs* Family of Genes Under Salt Stress

**Figure 6:**
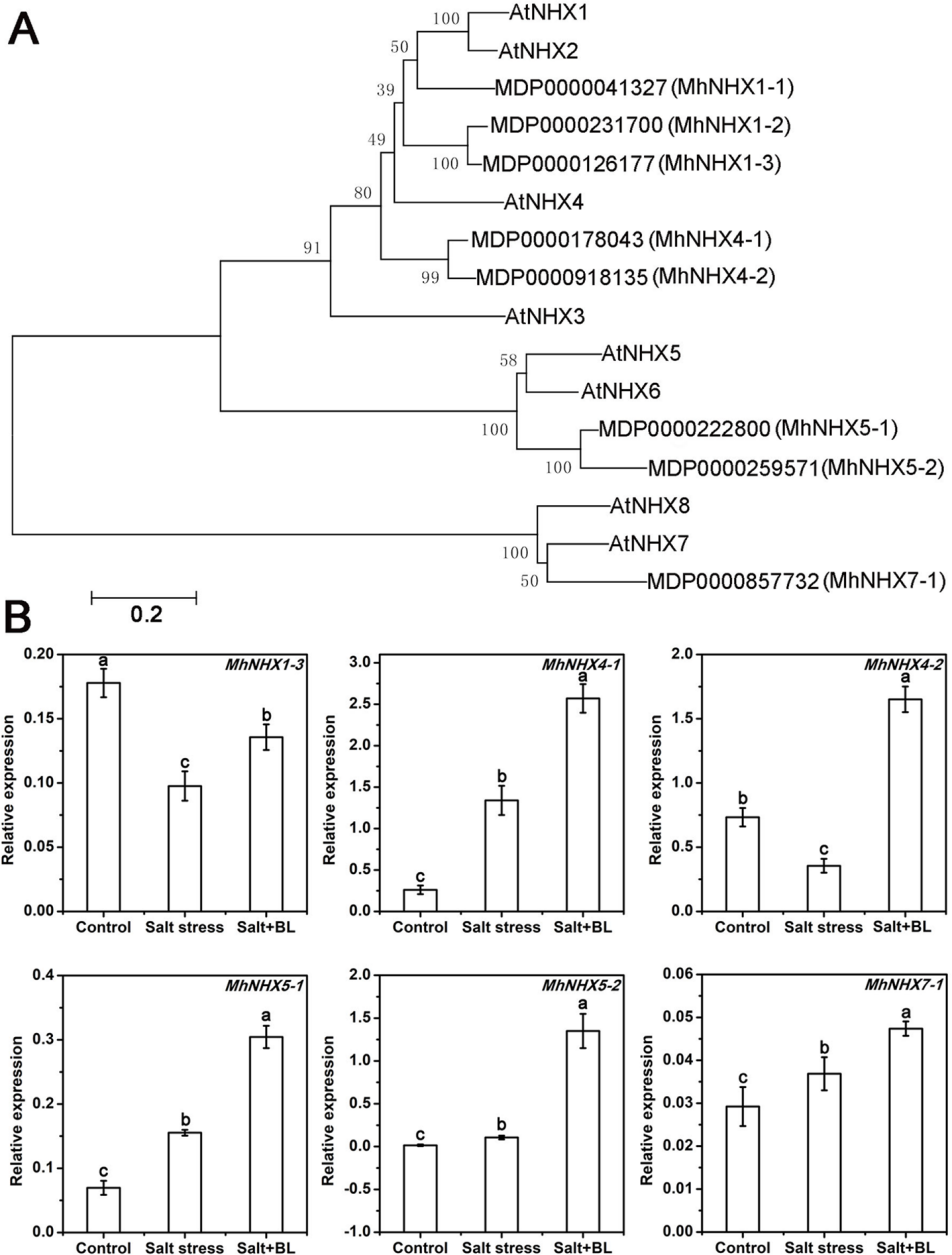
Phylogenetic tree of the amino acid sequences of the NHX family in apple and *Arabidopsis* and effects of exogenous BL application on the expression of *MhNHXs* family genes in apple seedlings under salt stress. **(A)** The phylogenetic tree was constructed using MEGA5.2 software with the neighbor-joining method and a bootstrap test with 1000 iterations based on the amino acid sequences of NHXs in apple and *Arabidopsis*. **(B)** The effects of exogenous BL application on the expression level of *MhNHXs* (*MhNHX1-3*, *MhNHX4-1*, *MhNHX4-2*, *MhNHX5-1*, *MhNHX5-2*, and *MhSOS1*) in apple seedlings under salt stress. Each experiment was independently repeated three times. Data represent the means ± SD of three biological replicates. Different lowercase letters indicate significant differences according to Fisher’s LSD (*P* < 0.05).

A phylogenetic tree was constructed according to the amino acid sequences of MhNHXs and AtNHXs. As shown in **Figure 6A**, five *MhNHXs* genes homologous with *AtNHX1*, *2*, *3*, and *4*; two *MhNHXs* genes homologous with *AtNHX5* and *6*; and the *MhNHX7-1* (*MhSOS1*) gene, which is homologous with *AtNHX7* (*AtSOS1*) and *AtNHX8* were observed.

The expression of the *MhNHXs* gene family was detected. The expression of *MhNHX4-1*, *MhNHX5-1*, *MhNHX5-2*, and *MhNHX7-1* was induced by salt stress, whereas the expression of *MhNHX1-3* and *MhNHX4-2* was suppressed by salt stress. Notably, the expression of *MhNHX1-3*, *MhNHX4-1*, *MhNHX4-2*, *MhNHX5-1*, *MhNHX5-2*, and *MhNHX7-1* was induced by exogenous BL (**Figure 6B**).

### Effects of Exogenous BL Application on the Expression of the *MhBZRs* Gene Family Under Salt Stress

**Figure 7:**
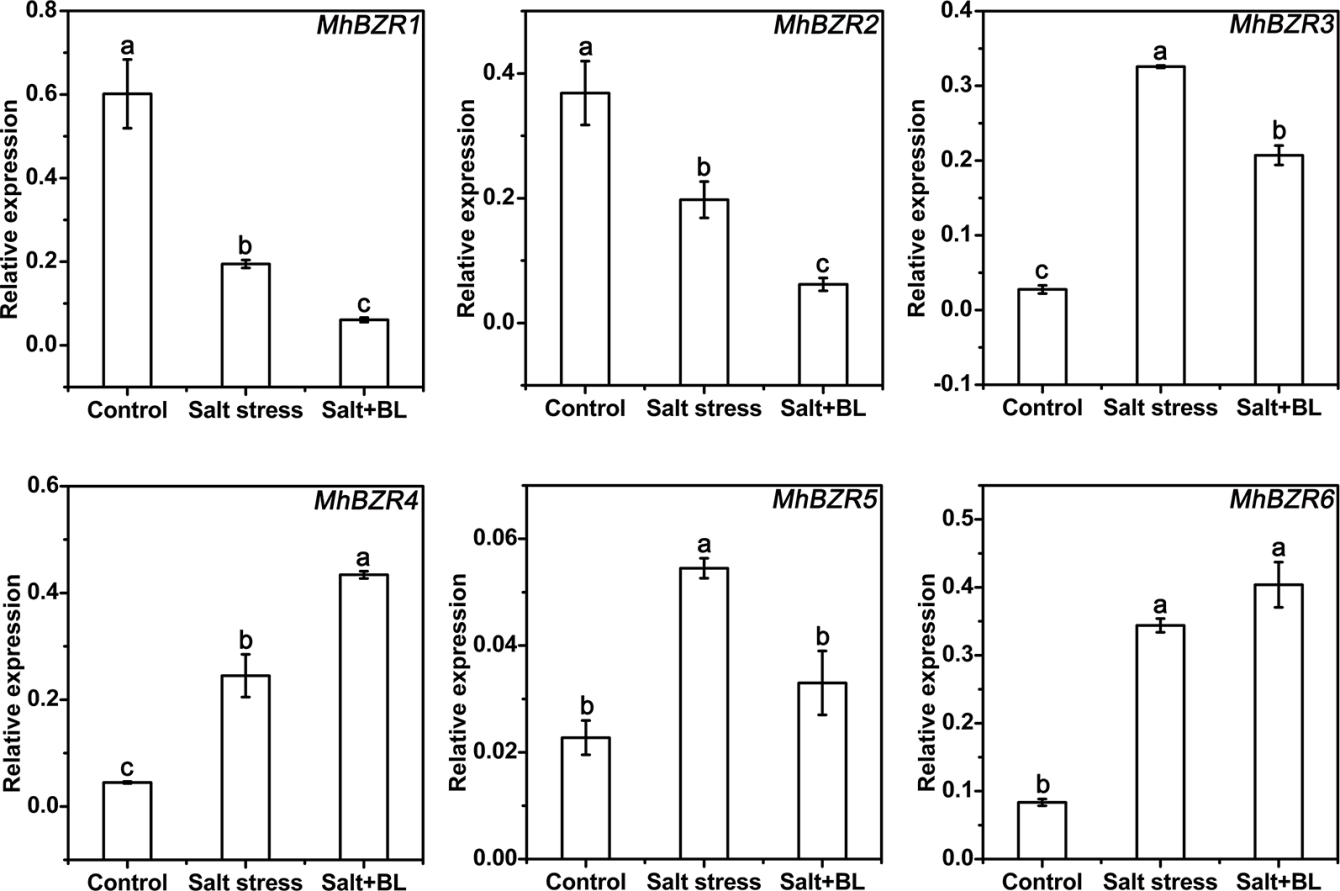
Effects of exogenous BL application on the expression levels of the *MhBZRs* gene family (*MhBZR1*, *MhBZR2*, *MhBZR3*, *MhBZR4*, *MhBZR5*, and *MhBZR6*) in apple seedlings under salt stress. Each experiment was independently repeated three times. Data represent the means ± SD of three biological replicates. Different lowercase letters indicate significant differences, according to Fisher’s LSD (*P* < 0.05).

Six *MhBZRs* genes that were homologous with *AtBZR1* and *AtBZR2* were identified. qPCR results showed that *MhBZR1* and *MhBZR2* were suppressed by salt stress, and *MhBZR3*, *4*, *5*, and *6* were induced by salt stress. When exogenous BL was applied under salt stress, the expression levels of *MhBZR1*, *2*, *3*, and *5* were suppressed, whereas that of *MhBZR4* was increased compared with the treatment without BL (**Figure 7** and **Figure S1**).

### MhBZR1 and MhBZR2 Directly Bind to the Promoter of *MhSOS1* or *MhNHX4-1 and* Repress Their Transcription

**Figure 8:**
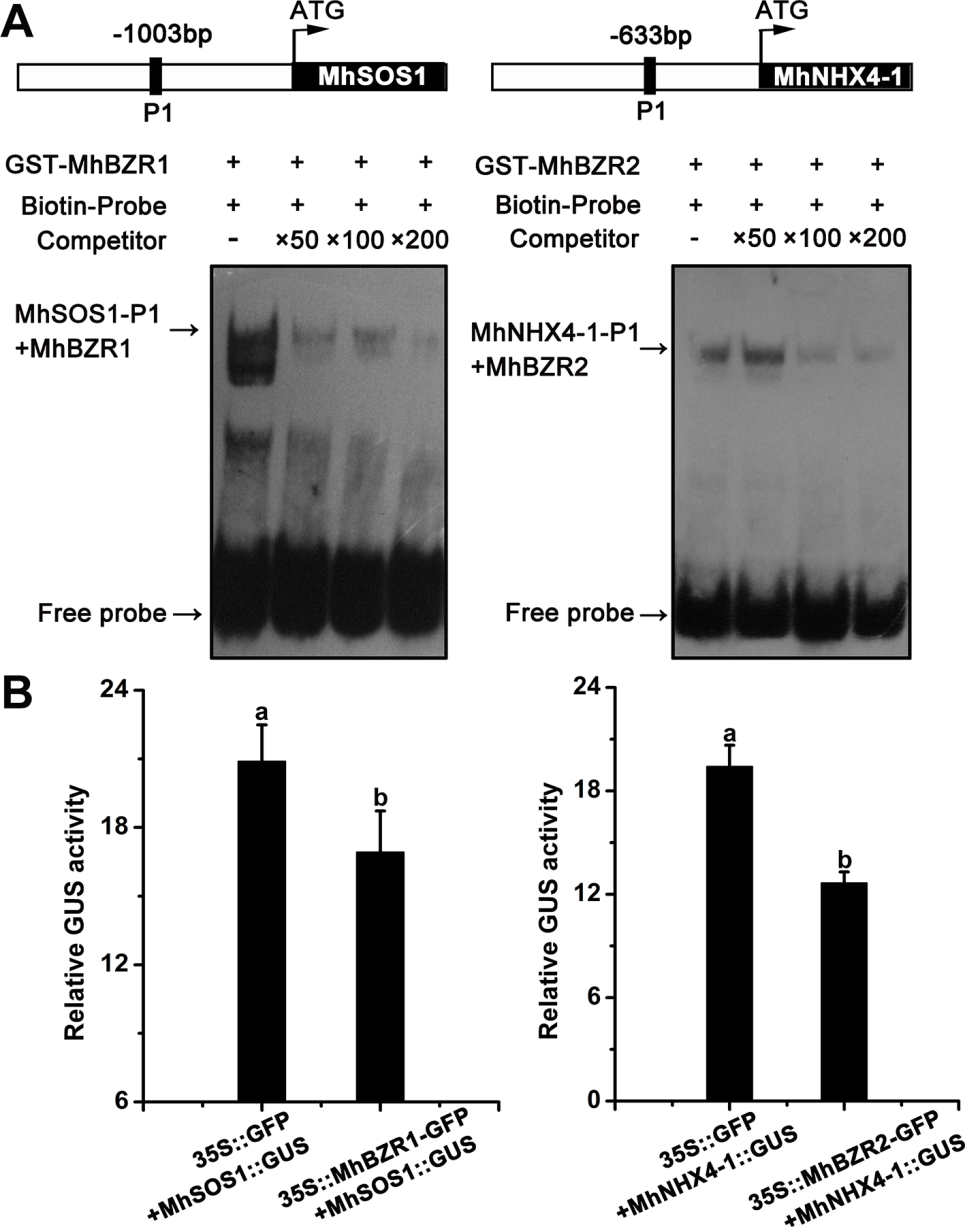
MhBZR1 and MhBZR2 bind to the promoter of *MhSOS1* or *MhNHX4-1* and inhibits their transcription, respectively. **(A)** Electrophoretic mobility shift assay (EMSA) results show the *in vitro* binding of MhBZR1 and MhBZR2 to the *MhSOS1* and *MhNHX4-1* promoters. The arrow indicates the position of a protein/DNA complex after the incubation of a biotin-labeled DNA probe and the GST-MhBZR1/MhBZR2 protein. Both the probes containing an E-box (CANNTG) was synthesized according to the sequence of the *MhSOS1*/*MhNHX4-1* promoter. **(B)** The relative GUS activity normalized to luciferase (LUC) activity in transiently transformed apple calli overexpressing *35S::MhBZR1/MhBZR2-GFP*, *proMhSOS1/MhNHX4-1::GUS* and *35S::LUC*; *35S::GFP*, *proMhSOS1/MhNHX4-1::GUS* and *35S::LUC* were used as controls. Each experiment was independently repeated three times. Data represent the means ± SD of three biological replicates. Different lowercase letters indicate significant differences, according to Fisher’s LSD (*P* < 0.05).

EMSA was performed to examine the binding of the promoter of *MhNHXs* by MhBZRs. MhBZR1 was bound to the *MhSOS1* promoter *in vitro*. The addition of 50×, 100×, and 200× unlabeled competitor reduced the detected binding of MhBZR1. Similar results confirmed that MhBZR2 can bind to the *MhNHX4-1* promoter *in vitro* (**Figure 8A**).

As shown in **Figure 8B**, the GUS : LUC activity ratio was significantly lower in transgenic apple calli expressing *proMhSOS1::GUS* and *35S::MhBZR1-GFP* than in transgenic apple calli expressing *proMhSOS1::GUS* and *35S::GFP*. Similar results were observed in *MhBZR2* to *MhNHX4-1*. Hence, MhBZR1 and MhBZR2 can directly bind to the promoter of *MhSOS1* and *MhNHX4-1* and repress their transcription.

## Discussion

Soil salinization negatively affects crop growth and is one of the major limiting factors of agricultural production (Zhu, 2016). Increased salinity in soil has severely hampered the development of apple plantations and resulted in economic losses for orchardists (Hu et al., 2018). The application of BL is a promising method for improving salt tolerance in plants (Shahzad et al., 2018; Tanveer et al., 2018). However, in woody plants, such as apple, the effect of exogenous BL application on seedlings under salt stress has not been reported. In our study, we applied different concentrations of BL to salt-stressed apple seedlings and found that the effects of BL application was much better at 0.2 mg/L than at 0.05 and 1.0 mg/L (**Figure 1**). Plant hormones are usually trace and efficient, and plant growth regulators affect plant growth and development usually in a dose-dependent manner. Low BL concentrations (0.1 and 0.01 µg/L) promote root elongation and lateral root development, whereas high BL concentrations (1–100 µg/L) inhibit root elongation (Hu et al., 2016b). Our result indicated that 0.2 mg/L BL would be an appropriate concentration for enhancing salt tolerance of *M. hupehensis* seedlings.

Salt toxicity to plants generally causes reduction in their photosynthetic performance due to the negative effects of salt stress on chlorophyll content and photosynthetic machinery (Wu et al., 2017). Wani et al. (2017) reported that salt stress inhibits the synthesis of 5-aminolaevulinic acid, a precursor of chlorophyll. Sadeghi and Shekafandeh (2014) reported the enhanced activity of chlorophyllase, the enzyme catalyzes the degradation of chlorophyll molecules. The present study demonstrated that salinity stress can significantly reduce the chlorophyll content of apple seedlings, and exogenous BL treatment can recover the content to normal levels (**Figure 2A**). The recovery of chlorophyll contents after the exogenous application of BL might be due to the modulation of transcription and translation processes by BR signaling, which ultimately stimulates chlorophyll biosynthesis and inhibits the breakdown of chlorophyll molecules (Sharma et al., 2016; Dong et al., 2017). Moreover, the present study indicated that the photosynthetic rate of the apple seedlings was reduced by salt stress, and exogenous BL application significantly improved photosynthetic rate under salt stress (**Figure 2B**). The application of BL enhances the leaf area of plants under salt stress (Sadeghi and Shekafandeh, 2014). In cucumber, BL increases photosynthesis by increasing chlorophyll content (Yuan et al., 2012). The application of BL improves photosynthesis by positively regulating synthesis and the activation of rubisco enzyme, thus increasing the gaseous exchange parameters of plants under salt stress (Fariduddin et al., 2014; Yusuf et al., 2017). It can be inferred that exogenous BL improves the photosynthetic rate of the apple seedlings under salt stress mainly by increasing chlorophyll content, leaf area, stomatal conductance, and rubisco enzyme activity.

Three main challenges are encountered by plants under salt stress, namely, osmotic stress, ion toxicity, and oxidative damage. Osmotic stress and ionic stress usually result in the oxidative damage with the accumulation of ROS, including O_2_·^−^ and H_2_O_2_ (Miller et al., 2010). In our study, the O_2_·^−^, H_2_O_2_, and MDA contents were sharply induced by salt stress, and exogenous BL application can markedly diminish them under salt stress (**Figure 3**). Exogenous BL application can reduce oxidative damage and decrease the production of ROS (Fariduddin et al., 2013; Lopez-Gomez et al., 2016). To prevent oxidative damages induced by salt stress, plants have developed an antioxidant system to maintain a dynamic balance of ROS. The antioxidant system comprises antioxidants and antioxidant enzymes. Enzymatic ROS scavenging mechanisms mainly include SOD, POD, and CAT (Ismail et al., 2014). The present study demonstrated that the accumulation of O_2_·^−^ and H_2_O_2_ and the increased concentration of MDA under salt stress were associated with the change of SOD, POD, and CAT activities. When exogenous BL was applied, the activities of SOD and CAT were significantly increased by 7% and 64%, respectively (**Figure 3**). The exogenous application of BL triggers the antioxidant enzyme systems of plants under salt stress, resulting in decreased oxidative stress (Tofighi et al., 2017). Wani et al. (2017) reported that exogenous BL application can increase the activities of SOD (75%) and CAT (52%) under salt stress in *Cicer arietinum*. This may be caused by that BL promotes the transcription and/or translation of the specific genes of antioxidant enzymes (Bajguz and Hayat, 2009). In addition, BL is also involved in the upregulation of genes enhancing the level of antioxidant enzymes subjected to salt stress (Cao et al., 2005; Ali et al., 2007).

Osmotic stress results from the effect of high salt concentrations in soil. Excess soluble salts in soil reduce the water potential at the root surface, which leads to water deficit in plants (Yang and Guo, 2018). Leaf electrolyte leakage is an important indicator of plant cell permeability. Our results indicated that electrolyte leakage was sharply induced by salt stress, and exogenous BL can decrease electrolyte leakage and protect apple seedlings from osmotic stress (**Figure 4A**). Under salt stress, plants accumulate various osmolytes in the cytoplasm, including soluble sugar and proline, through which they sustain their osmotic equilibrium and support the absorption of water (Liang et al., 2018). Our experiments revealed that salt stress increased the contents of proline, soluble sugar, and soluble protein. The application of exogenous BL enhanced the contents of proline and soluble sugar but did not influence the levels of soluble protein (**Figure 4**). In *Triticum aestivum*, a significant increase in proline content and soluble sugar was noticed after the application of BL, thus protecting plants from the adverse effects of salt toxicity (Talaat and Shawky, 2012; Yusuf et al., 2017). Our results indicated that exogenous BL can protect apple seedlings from osmotic stress mainly through the accumulation of proline and soluble sugar.

The primary challenge for plants under salt stress is ionic toxicity, which is caused by the excessive accumulations of sodium in the cytoplasm. This ionic toxicity can lead to an imbalance in cytosolic Na^+^:K^+^ ratio and disrupt normal plant growth (Dai et al., 2018). In the present study, Na^+^ and K^+^ contents in the shoots were increased under salt stress. When exogenous BL was applied to the apple leaves, Na^+^ content decreased with increased K^+^ content, and thus the leaf Na^+^/K^+^ ratio decreased (**Figure 5**). This condition is consistent with the findings of Sun et al. (2015b) in which 10 nM BL treatment reduced leaf Na^+^/K^+^ and increased K^+^ content in *perennial ryegrass* under salt stress. However, in the roots, exogenous BL notably increased K^+^ content. Given that exogenous BL was applied to the leaves, we inferred that the increased K^+^ content in roots was caused by the transportation and distribution of K^+^ between shoots and roots, which was affected by exogenous BL to the leaves. Recently, Azhar et al. (2017) showed that BL in the growth medium prevented salt-induced K^+^ leak from shoots and roots. They also noted that BL decreased K^+^ efflux under salt stress by controlling depolarization-activated GORK channels. Our results indicated that exogenous BL can enhance the exclusion of Na^+^ and increase the absorption of K^+^ both in shoots and roots under salt stress.

The physiological mechanism of exogenous BL on plants has been widely explored. However, limited information is available regarding the transcriptional changes and the molecular mechanism in response to BL under salt stress. Na^+^(K^+^)/H^+^ antiporters (NHX) are essential compounds and are critical for modulating plant Na^+^:K^+^ homeostasis (Carden et al., 2003). The overexpression of apple *MdSOS1* can enhance salt tolerance and reduce the content of Na^+^ (Liu et al., 2012). In the present study, *MhSOS1* was induced by salt stress and enhanced by exogenous BL treatment. Thus, the decrease in Na^+^ content following exogenous BL treatment under salt stress was mainly caused by the induced expression level of *MhSOS1*. Furthermore, AtNHX1, 2, 3, and 4 are located in vacuoles and regulate K^+^ homeostasis between the cytoplasm and vacuoles (Craig Plett and Moller, 2010; Barragan et al., 2012). In apple, the overexpression of *MdNHX1* significantly enhances salt tolerance (Li et al., 2010). Our results showed that *MhNHX1-3*, *MhNHX4-1*, and *MhNHX4-2* expression levels were increased by exogenous BL treatment (**Figure 6**). We assumed that exogenous BL application increased K^+^ content mainly through the regulation of transcriptional changes of these genes.

BR regulates a wide spectrum of cellular activities and biological processes, which depend, at least in part, on the direct regulation of gene expression *via* the BZR1/BES1 family of transcription factors, thus eliciting various BR responses. BZR1 and BES1 are the key transcription factors in the BR signaling pathway that directly regulates the expression of many target genes (Sun et al., 2010; Kim et al., 2017). Information about the transcriptional regulation of the NHX family is still limited. The sugar beet gene *BvNHX1* is regulated by the MYB transcription factor (Diedhiou et al., 2009; Adler et al., 2010). We assume that exogenous BL application can regulate transcriptional changes in *MhNHXs* through *MhBZRs*. In our study, EMSA results indicated that *in vitro*, MhBZR1 and MhBZR2 can directly bind to the E-box of the *MhSOS1* or *MhNHX4-1* promoter, respectively (**Figure 8A**). The transiently transformed calli expressing the GUS reporter indicated that *in vivo*, MhBZR1 and MhBZR2 can repress the expression of *MhSOS1* and *MhNHX4-1*, respectively (**Figure 8B**). Overall, our study revealed the partial molecular mechanism of the effect of exogenous BL application on salt stress in apple.

## Data Availability Statement

The datasets generated for this study are available on request to the corresponding author.

## Author Contributions

CW and YT planned and designed the research. QS and XZ performed experiments, conducted fieldwork, analyzed data, etc. QS, XZ, and CW wrote the manuscript.

## Funding

This study was supported by the Funds for Modern Agricultural Industry Technology System in Shandong Province, China (SDAIT-06-06) and Shandong Provincial Natural Science Foundation, China (ZR2019BC038).

## Conflict of Interest

The authors declare that the research was conducted in the absence of any commercial or financial relationships that could be construed as a potential conflict of interest.
