# Supplementary material for: Exogenous Brassinolide Alleviates Salt Stress in Malus hupehensis Rehd. by Regulating the Transcription of NHX-Type Na+(K+)/H+ Antiporters
Source: Front Plant Sci. 2020 Feb 6;11:38. doi: 10.3389/fpls.2020.00038 (PMC7016215; doi:10.3389/fpls.2020.00038)
Supplement: Supplementary file 1 [file DataSheet_1.pdf]

Supplementary Material

Exogenous Brassinolide Alleviates Salt Stress in *Malus hupehensis* Rehd. by Regulating the Transcription of NHX-type $Na^{+}(K^{+})/H^{+}$ Antiporters

Qiufang Su, Xiaodong Zheng, Yike Tian, Caihong Wang*

***Correspondence: Caihong Wang**: chw6068@126.com

1. Supplementary Figures

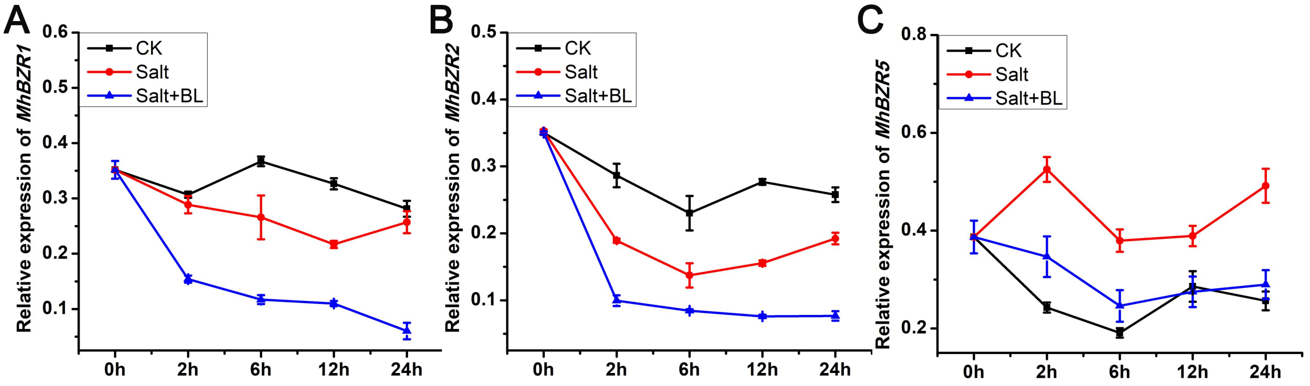

Supplementary Figure 1. Effects of exogenous BL application on the expression level of the *MhBZR1*, *MhBZR2* and *MhBZR5* in 24h under salt stress. Data represent the means ± SD of triplicate experiments.

2. Supplementary Tables

Table 1 The primers used for cloning, vector construction and qRT-PCR

Primer name	Forward primer	Reverse primer	Vector
MhNHX4-1 promoter	TAAACCGGCAGTTGTTGTCAGTTGCCCAATTGGCGGGGAA	TTCCCCGCCAATTGGGCAACTGACAACAACTGCCGGTTTA	
MhSOS1 promoter	TTACGTGTGACTGGGAAGTTGGCTCCTTGCACATATGTTC	GAACATATGTGCAAGGAGCCAACTTCCCAGTCACACGTAA	
MhBZR1 for cloning	ATGACGTCGGATGGGGCG	TTAAATCCGAGCCTTTCCA	
MhBZR2 for cloning	ATGACGTCTGACGGAGCAA	CATTGGCCGGAATCTGC	
MhBZR3 for cloning	ATGACAGGCGGTGGTTCAT	TCAAAACCCAGCTGGGA	
MhBZR4 for cloning	ATGACGTCGGGGACGCGA	TCAGAAATAACGAGTTCGC	
MhBZR5for cloning	ATGATGACGTCGGGAACG	ACCTGGTCCGAGTGCTCC	
MhBZR6 for cloning	ATGACGTCGGGAACCAGA	TTACCTYGTTCGAGTGCTC	
MhBZR1-pGEX6P -1	GGATCCATGACGTCGGATGGGGCG	CTCGAGTTAAATCCGAGCCTTTCCA	pGEX6P -1
MhBZR2-pGEX6P -1	GGATCCATGACGTCTGACGGAGCAA	CTCGAGCATTGGCCGGAATCTGC	pGEX6P -1
MhBZR3-pGEX6P -1	GGATCCATGACAGGCGGTGGTTCAT	CTCGAGTCAAAACCCAGCTGGGA	pGEX6P -1
MhBZR4-pGEX6P -1	GAATTCATGACGTCGGGGACGCGA	CTCGAG TCAGAAATAACGAGTTCGC	pGEX6P -1
MhBZR5-pGEX6P -1	GGATCCATGATGACGTCGGGAACG	CTCGAGACCTGGTCCGAGTGCTCC	pGEX6P -1
MhBZR6-pGEX6P -1	GGATCCATGACGTCGGGAACCAGA	GAATTCTTACCTYGTTCGAGTGCTC	pGEX6P -1
MhNHX1-3-qPCR	GCTTATGCGTGGCTCTGTTTC	CGGGTCCTATGTTTGCCTCTG	
MhNHX4-1-qPCR	CATACTCAACAGCGTGGAAACG	TAAGGAACGAACCCTCTACCG	
MhNHX4-2-qPCR	GGATGCCTTGGACATTGAGAA	AAGCAAGAGCCACGGATACAG	

MhNHX5-1-qPCR	TCAAAGGTAGTCGGGGGTAGAG	TTTCCGTATTAGAGATGTTAGCAAGT
MhNHX5-2-qPCR	AGTCAAACGCCAAGCAGCAC	ATGGTGGGACTGGAGGAAGG
MhSOS1-qPCR	TCCGGTTAATCCATCACACACCGT	TTTGCTGCCCTGGAGGATTTGTTG
MhBZR1-qPCR	CCAAGCACTGCGACAACAA	GACTGGGATAGGACGAAGACG
MhBZR2-qPCR	CCCAAGCACTGCGACAACA	GGATAGCACGAAGACGAAGGAC
MhBZR3-qPCR	GAGGATGGCACCACCTACCG	CTGGGATTTCAACAAGAAAGCAAG
MhBZR4-qPCR	TCCACAGCGGCTCTATCAGT	TCGGAATCCCAGCAAACCAT
MhBZR5-qPCR	GGGCAGCATTACCCTTTCCT	CGAGATCATCTGATACGCATTCC
MhBZR6-qPCR	TAAGCACTGCGACAACAACG	CCATCACAATCTCAAACCCAC
MhActin	CTTCAATGTGCCTGCCATGTAT	AATTTCCCGTTCAGCAGTAGTG
